# Wounds of the Fingers, and How to Treat Them

**Published:** 1885-06

**Authors:** John Kent Spender

**Affiliations:** Physician to the Mineral Water Hospital, Bath.


THE BRISTOL

Medico-Chirurgical Journal.

JUNE, 1885.

### WOUNDS OF THE FINGERS, AND HOW TO TREAT THEM.

BY

JOHN KENT SPENDER, M.D. Lond.,

*Physician to the Mineral Water Hospital, Bath.*

---

SOME of the minor casualties which come within the range of surgical practice require quick and ready help; such help, I mean, as ought to be within the capacity of every medical man, whatever may be his qualifications or the technical method of exercising his craft. As old as Ulysses is the song which celebrates the wise physician, "skilled our wounds to heal;" and the conventional distinctions of our own day deserve a hearty ridicule if they prevent a Doctor of Medicine from relieving a distended bladder, stopping a dangerous hæmorrhage, or putting a little plaister over a gaping wound. To relieve pain and to repair an injury are the noblest badges of our art. These functions belong to no special bureau; they are the common aims of all medico-chirurgical science; and he who can most expertly do this twofold work comes nearest to the ideal standard of a medical practitioner.

The hand is the most finished symbol of a human creature,* and the fingers are the terminal instruments by which most of the powers and utilities of the hand are carried out. Injuries and deformities of the fingers may, therefore, spoil and even permanently cripple the dexterity of the whole upper limb. The pathological anatomy of the fingers has not received the attention which it merits. An irregular brigade of busy folk, called chiropodists, ply their trade on the toes with no little success; and all sensible doctors welcome the aid of these unlicensed auxiliaries, for their work demands special tools, and also certain petty aptitudes which are a sort of natural gift. But who takes care of the fingers? Chilblains infest them; warts disfigure them; they are weakened by rheumatism and gout; whitlows burrow among the tendons and fascia; and the diseases of the nails are a sufficient study for the dermatologist.† But if the medical troubles of the fingers are within the territory of both physician and surgeon, I plead that sudden traumatic disasters should belong to the same territory too;

* The finest English monograph on the human hand, from a philosophical standpoint, is the Bridgewater Treatise by Sir Charles Bell. The argument is well developed; that argument, I mean, which was the speciality of the Bridgewater Treatises. During a botanical excursion from King's College in 1849 or 1850, I heard the late Professor Edward Forbes say that he greatly disliked what he called "Bridgewater Treatising." Perhaps the logic of those once famous volumes was not always coherent or consistent; and one of its dangers lies in evoking the kind of challenge lately put forward by a distinguished oculist, who maintains that the weaknesses and imperfections of the human eye prove that an all-wise Creator could not have created it. (See an astonishing page in the *Practitioner*, January, 1882, p. 70.)

† One of the plates of the New Sydenham Society's *Atlas* may be consulted.

for a trifling mutilation of only one finger may cause life-long deformity and disability, and be an abiding witness of neglect or want of skill.

The text of my paper is a case which occurred to me last summer, and which shall be related in the fewest words.

On the morning of August 1st, 1884, the son (aged 22) of a tradesman was brought to my house with his left hand bleeding profusely. Half an hour before, the third and little fingers had been caught and cut by some machinery; in both fingers the cut had gone across the matrix of the nail; the last phalanx of the third finger was almost separated; in the little finger the wound was not so deep. I stanched the flow of blood with circular pledgets of lint; next I fastened the arm and hand to a board, and suspended the whole limb in a sling; and the last step of these preliminary proceedings was to send the patient home to recover from the shock, with the help of warm food and a little sleep. Four hours afterwards I visited him, and dressed the injured fingers in the following way:

Firstly, the pledgets of lint, stiffened with dry blood, were soaked in water and gently removed. No foreign body of any kind was found. The fingers were thoroughly cleansed, the nearly separated portions were brought into juxtaposition, and retained *in situ* while a circlet of boric lint was applied. Each finger was laid upon a bed of absorbent cotton-wool tissue, which just met on the dorsal side; then a Lilliputian bandage of thin soft calico was put around, with moderate pressure, and the turns of bandage were brushed over with the gum acacia mucilage of the *British Pharmacopœia*. Finally, each finger was put into a cradle of gummed paper, which was moulded while soft, and then dried in the gradual heat of an oven. These light and simple shields kept the fingers apart, and guarded them from further accident.

It will be observed that, with the exception of the boric lint, not a scrap of antiseptic material was used. The two aims of the mechanical treatment, were (*a*) to render the injured parts absolutely immobile, and (*b*) to keep off anything which might hurt them, whether that "anything" were invisible morbific germs, or coarse physical roughness. In doing the former work, the latter was done too: the very machinery devised for the one purpose fulfilled the other. Perfect immobility could be obtained only by an apparatus which surrounded every phalanx and every joint; flexor and extensor tendons were held in bonds which they could not burst; and the completeness of these bonds shut out all air, with its legion of spores, dust, and noxious gases. The common air which we all breathe, whether we like it or not, is said to be so full of infective stuff and contagious mischief that it is a physical wonder how anybody lives at all, and a still greater wonder that there is any enjoyment and glory in life. Even the purest medical atmosphere is, in a surgical sense, a vile medium, microscopically loathsome; and to keep it at a distance is one of the cunning stratagems of surgical art. The question, therefore, is this: If a wound be sealed all round with a wrapping which is impermeable to air, is not this sufficient in itself to keep the wound sweet and non-purulent until it is healed? Or must the wrapping be charged with a so-called antiseptic quality?

I venture to say that it is the exclusion of the germ-bearing air which is the secret of the whole matter. We are not discussing now surgical wounds made for ulterior purposes, such as the extraction of tumours, the letting-out of fluid, or examining the condition of any of the viscera: these "vivisections" are made with deliberation and science, and are very properly hedged round with the safeguards of antiseptic weapons and an antiseptic atmosphere. But the unforeseen and sudden wound which draws blood, done in a moment, and being nought but evil in its suffering and its consequences, ought to be closed, wrapped up, and made a "sealed book" as quickly as possible. And if not so quickly as it ought to be, we have at least done our part as soon as we can; and my contention is, that the conditions of the problem are fulfilled if the severed flesh be brought together, and then locked up (so to speak) until it be healed. Where was the superstition born, and how was it bred, that a wound needs to be incessantly meddled with, looked at, sponged, sprayed, and finally covered with some stinking acrid stuff which is said to kill germs? Do we take up a tree every few hours to see how it grows?

It is time to return to the case which is the text of my paper. On the following day my young patient said that he had slept as usual, and he seemed free from all pyrexial disturbance. The anxious domestic question was this: The parents had arranged their plans for a holiday of three days (including the Bank Holiday of August 4th): might the son go with them to the seaside? I saw no objection, and when he returned on Wednesday morning, the 6th, the report was that the fingers were "very comfortable;" there was no swelling or pain in the hand and arm; everything seemed sweet and pure, and the apparatus was in perfect order. On the same evening I left home for my usual holiday, and transferred the case to my friend, Mr. Hopkins. The wounded fingers were not undressed until eleven days had elapsed from the date of the accident;* and when exposed to view the healing was found to be complete, and the fingers were of their natural size (though, of course, a little shorter than before the injury). The tender cicatrices were protected for a few more days with ordinary plaister, and the work was finished.

Mr. Sampson Gamgee is the latest prophet and missionary of John Hunter's "epoch-making generalisation," that absolute immobility is requisite for the restoration of wounds and fractures. Immobility is another name for local rest; *local* in contradistinction to that general rest which is often medically injurious, because so disturbing to physiological functions. Immobility, in a surgical sense, may be attained by uniform compression over a large area; a compression which has no therapeutic alliance with constriction, for this implies a tight fetter over a narrow band of surface, squeezing nerves, and obstructing blood-vessels. Mr. Gamgee's book, *On the Treatment of Wounds and Fractures*, is an eloquent exposition of this important principle of surgical therapeusis—a principle which is far-reaching in its application, and wonderfully helpful to the comfort of our patients. The instruments of Mr. Gamgee's workmanship are cotton absorbent gauze, softened millboard splints, and thin calico bandages. These are welded together into an uniform mass by the aid of liquid starch; muscles are rested, bones are supported, joints are immobilised, and textural damage of every kind quietly mends. Nothing else but mending is, indeed, possible, so long as a part has any life in it. Buried from all sight and harm, interred within a rigid cuirass of benign swathings, the

* During all this time the weather was very hot.

marvel would be if wound, fracture, or strain did not obey that natural order which, when we do not thwart it, always strives to bring right out of wrong.

The exceeding simplicity of Mr. Gamgee's method is a high commendation to the reasonable mind; for art is most simple when it tries to imitate Nature. And if our efforts should be baffled and altogether beaten, then, and then only, are we entitled to speak of hidden dynamic forces stopping the way; it may be embolism, or arterial obstruction, or poisoned blood, or bruised and shaken nerves. We deserve to fail when we not only do not imitate Nature, but do some thing which defies her "first principles," and disobeys those lessons which may be learnt at the bedside whenever we choose to sit down and learn them.

In the summer of 1870 I met, in a remote part of a neighbouring county, a skilful surgeon in consultation. A labouring man, while hewing a tree, had plunged a hatchet into his knee-joint, and thereby made a large and dangerous wound. The man's mistress, a benevolent lady, who had been lately under my care, desired to have my advice in the matter. We acted in accordance with the then authentic practice in putting the injured joint under a dropping cascade of the coldest water; special safeguards were provided against movement and the admission of air into the cavity of the joint, and bags of ice were sometimes placed upon it. Opium was freely given, and plenty of nourishment. Of course the man died, and there were plenty of reasons why he could not do otherwise. Now, what would be done in such a case by the pure restorative surgery of to-day? The wound would be covered by lint, dipped in a styptic colloid; its vital temperature would be maintained by an environment of dry wool or wadding; immobility would be secured by millboard splints and bandages; and vascular tension would be reduced by steady elevation of the limb. Given a fairly healthy patient, and our success is almost certain. It is in wounds of the joints that Mr. Gamgee most clearly demonstrates the truth of his principles and the facility of their application. His cases exhibit a remarkable success, and it is interesting to note how his practice was foreshadowed by William Hey and the elder Larrey.

The fingers are tiny organs, but they are large enough to be landmarks for sound practice, and beacons against bad. There is room to try many surgical schemes on these tapering little cylinders of flesh and blood; and the treatment of other injuries; besides incised wounds, requires to be carefully weighed and dexterously carried out. The doctrines of Hilton and Lister can never grow old, and their importance is clear to the most slender understanding. But what those doctrines really imply is not always so clearly seen. During the process of healing, *rest* means equal support in all directions, and *antisepticism* means absolute protection from every soil of this "work-a-day world." Attend to the conditions which these terms convey, and an insignificant injury will not have its dark shadows of abscess, or erysipelas, or gangrene. In the petty "operation" of dressing a cut finger, do everything with as much neatness and precision as if you were dealing with a deep stab in the chest, or with the manifold lesions of a compound fracture. And if we are apt scholars in the school of prevention, as well as in the school of curing, we shall less often exemplify the cleverness of which Goldsmith sings in his famous elegy to Mrs. Mary Blaize, who died, and then—alas! too late—

"The doctors found, when she was dead,
Her last disorder mortal."

